# Climatic Diversity and Ecological Descriptors of Wild Tomato Species (*Solanum* sect*. Lycopersicon*) and Close Related Species (*Solanum* sect*. Juglandifolia* y sect. *Lycopersicoides*) in Latin America

**DOI:** 10.3390/plants10050855

**Published:** 2021-04-23

**Authors:** Gabriela Ramírez-Ojeda, Iris E. Peralta, Eduardo Rodríguez-Guzmán, José Luis Chávez-Servia, Jaime Sahagún-Castellanos, Juan Enrique Rodríguez-Pérez

**Affiliations:** 1 Departamento de Fitotecnia, Universidad Autónoma Chapingo (UACh), Chapingo 56230, Mexico; gabramirezo@gmail.com (G.R.-O.); jsahagunc@yahoo.com.mx (J.S.-C.); 2 Facultad de Ciencias Agrarias, Universidad Nacional del Cuyo (UNCUYO), Mendoza M5502JMA, Argentina; iperalta@fca.uncu.edu.ar; 3 Centro Científico Tecnológico CONICET, Instituto Argentino de Investigaciones de las Zonas Áridas, Mendoza C1425FQB, Argentina; 4 Centro Universitario de Ciencias Biológicas y Agropecuarias, Universidad de Guadalajara (UdG), Zapopan 45200, Mexico; edrg@hotmail.com; 5 Centro Interdisciplinario de Investigación para el Desarrollo Integral Regional Unidad Oaxaca, Instituto Politécnico Nacional (IPN) Xoxocotlán, Oaxaca 71230, Mexico; jchavezservia1@yahoo.com

**Keywords:** wild tomato species, ecological descriptors, environmental amplitude, climatic diversity, genetic resources

## Abstract

Conservation and sustainable use of species diversity require a description of the environment where they develop. The objectives were to determine ecological descriptors and climatic diversity of areas along the distribution range of 12 species of wild tomatoes (*Solanum* sect. *Lycopersicon*) and four wild species of phylogenetically related groups (*Solanum* sect. *Juglandifolia* and sect. *Lycopersicoides*), as well as their ecological similarity in Latin America. With 4228 selected tomato accessions and an environmental information system (EIS) composed of 21 climatic variables, diversity patterns of the distribution areas were identified for each species, as well as ecological descriptors through the use of geographic information systems (GIS). The contribution of climatic variables to the species geographical distribution was identified by principal component analysis (PCA), and similarity in species distribution as a function of the variables identified with cluster analysis (CA). Climatic characteristics and the environmental amplitude of wild tomatoes and related species along their distributional range were satisfactorily determined by ecological descriptors. Eleven climate types were identified, predominantly BSk (arid, steppe, cold), BWh (arid, desert, hot), and Cfb (temperate, no dry season, warm summer). PCA determined 10 most important variables were the most important for the geographical distribution. Six groups of species were identified according to CA and climatic distribution similarity. This approach has shown promissory applications for biodiversity conservation of valuable genetic resources for tomato crop breeding.

## 1. Introduction

Tomato (*Solanum lycopersium* L.), a member of the *Solanaceae* family, is one of the world’s leading vegetable crops with worldwide distribution growing in an extensive variety of habitats [1]. Peru has been considered the center of origin, but it is accepted that the tomato diversification process involved two transitions; the first occurred in South America, from the wild species *Solanum pimpinellifolium* L. to a partially domesticated species *Solanum lycopersicum* L. var. *cerasiforme* (SLC); while the second occurred in Mesoamerica from SLC to the completely domesticated species *Solanum lycopersicum* L. var. *lycopersicum*. However, Razifard et al. [2] recently reported that the origin of SLC may be prior to its domestication since many typical characteristics of tomatoes grown in South America come from this species; later, SLC was lost or diminished once the partially domesticated forms extended toward the north. Further strong artificial selection after the tomato was first introduced to Europe in the XVI century and in modern times greatly reduced the genetic variation of the crop [3]. Wild tomato species related to cultivated tomatoes are valuable resources because they provide genetic diversity due to their ecological adaptation. In addition to the cultivated species, there are 12 species of wild tomatoes (*Solanum* sect. *Lycopersicon* (Mill.) Wettst), and four wild species from two phylogenetically related groups (*Solanum* sect. *Juglandifolia* (Rydb.) A. Child and *Solanum* sect. *Lycopersicoides* (A. Child) Peralta). Wild tomato species of the *Lycopersicon* section are: *Solanum arcanum* Peralta, *Solanum cheesmaniae* (L. Riley) Fosberg, *Solanum chilense* Dunal, *Solanum chmielewskii* (C. M. Rick, Kesicki, Fobes, and M. Holle), D. M. Spooner, G. J. Anderson and R.K. Jansen, *Solanum corneliomulleri* J. F. Macbride, *Solanum galapagense* S.C. Darwin and Peralta, *Solanum habrochaites* S. Knapp and D. M. Spooner, *Solanum huaylasense* Peralta, *Solanum neorickii* D. M. Spooner, G. J. Anderson and R. K. Jansen, *Solanum pennellii* Correll, *Solanum peruvianum* L. and *S. pimpinellifolium* [1], and from the *Junglandifolia* and *Lycopersicoides* sections are *Solanum juglandifolium* Dunal, *Solanum lycopersicoides* Dunal, *Solanum ochranthum* Dunal, and *Solanum sitiens* I. M. Johnston [1,4,5].

A comprehensive treatment of wild tomatoes and close relatives, combining different evidence and their phylogenetic relationships, led to the proposed new classification proposed by Peralta et. al. [1]. The predictability of this classification has been verified in recent studies based on different molecular, genomic, and transcriptomic data of wild tomatoes [3,6,7]. The work of Peralta et. al. [1] has been considered to compare diversity climatic patterns and ecological descriptors. Wild tomatoes, Section Lycopersicon: “Lycopersicon group” (*S. pimpinellifolium*, *S. cheesmaniae* and *S. galapagense*), “Arcanum group” (*S. arcanum*, *S. chmielewskii* and *S. neorickii*), “Eriopersicon group” (*S. habrochaites*, *S. huaylasense*, *S. corneliomulleri*, *S. peruvianum,* and *S. chilense*), “Neolycopersicon group” (*S. pennellii*); outgroup close related species in Section Juglandifolia (*S. juglandifolium* and *S. ochranthum*) and Section Lycopersioides (*S. lycopersicoides* and *S. sitiens*).

Species of *Lycopersicoides* and *Lycopersicon* sections are generally plants that grow in dry habitats along the Pacific coastal range and in inter-Andean valleys, while species of *Junglandifolia* section are distributed in cloudy forests and open areas with high light intensity. Unlike wild species, cultivated tomato is more dependent on humidity and is located in disturbed sites from the tropics, subtropics to temperate zones with defined summers [1].

Wild tomatoes are generally annual; they germinate, grow, reach flowering, fructification, and die in a growing season, but if there is continuous humidity, they manifest as perennials. In other regions, they rarely persist for generations if agronomic management is not provided [1,8].

Breeding programs generally use a limited genetic base, so it is extremely important to explore and know the sources of natural variation [9,10] for identification of genes associated with more rustic characteristics such as resistance to drought [11], extreme temperatures [12], and resistance to pests and diseases [13,14], among others. Likewise, the characteristics of the organoleptic and nutraceutical quality of the fruit have gained importance in the improvement, characteristics that can be found in some wild populations [15,16].

There are various sources of information on the geographical location of tomato species and their gene pools, ex situ and in situ conservation programs, and germplasm banks [17,18,19,20]. In addition, some research has been carried out to identify the geographical and ecological patterns of some species [1,21,22,23]. However, the information on all the wild and related tomato species is scarce since the ecological descriptors and the particular climatic characteristics in which they are distributed are unknown or limited.

Ecogeographic studies of plant genetic resources allow the identification of the adaptive ranges of the species and the most relevant environmental variables that define their distribution [24]. Its main applications are related to the collection, conservation, characterization, documentation, and use of plant genetic resources [7,24,25,26,27]. Additionally, it is possible to predict the environmental conditions of the collection sites [11,28,29] from the ecological descriptors derived from the geographical location of germplasm and environmental variables obtained through GIS tools [10,11,28,29,30].

The central hypothesis of this research postulate that patterns of climatic diversity might coincide with the classification of wild tomatoes reflecting close ancestral relationships.

The objectives were determining the ecological descriptors and the climatic diversity of 16 species (12 species of wild tomatoes (*Solanum* sect. *Lycopersicon*) and four species of phylogenetically related groups (*Solanum* sect. *Juglandifolia* and sect. *Lycopersicoides*)), as well as their ecological similarity in Latin America.

## 2. Results

### 2.1. Climatic Diversity

**Figure 1:**
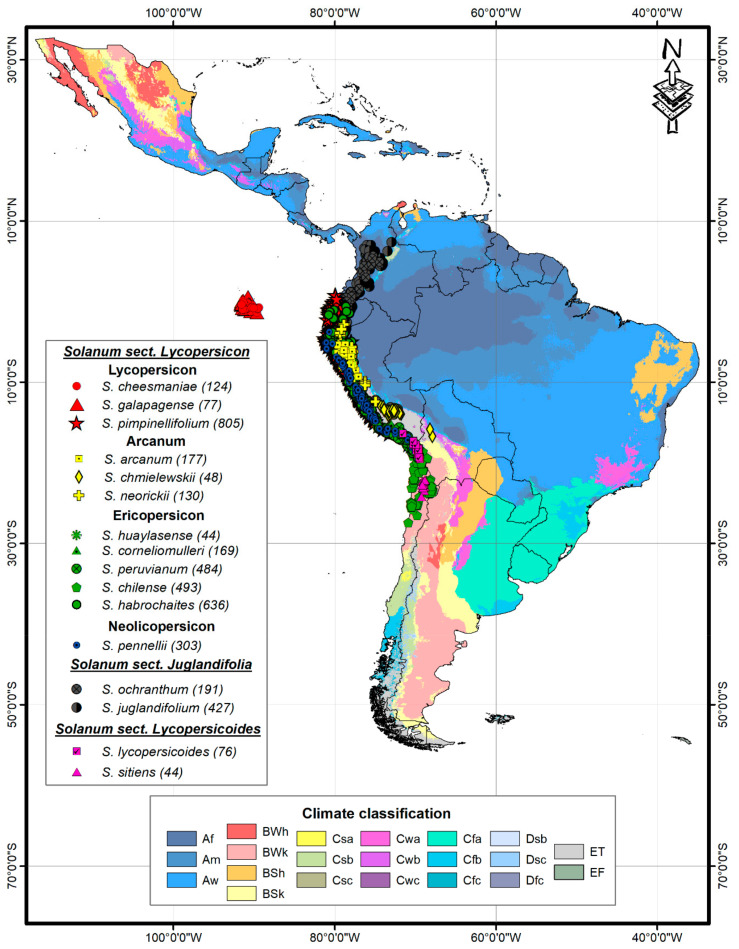
Climate classification according to Beck et al. [31] and geographic distribution of 12 wild tomatoes (Sect. Lycopersicon) and 4 closely related species (Sect. Juglandifolia and Lycopersicoides) Climate classification: Af (tropical, rainforest), Am (tropical, monsoon), Aw (tropical, savannah), BWh (arid, desert, hot), BWk (arid, desert, cold), BSh (arid, steppe, hot), BSk (arid, steppe, cold), Csb (temperate, dry summer, warm summer), Cwb (temperate, dry winter, warm summer), Cfb (temperate, no dry season, warm summer), and ET (polar, frost). In parentheses number of accessions.

Of the 21 existing climates in Latin America, according to the Köpen-Geiger classification adapted by Beck et al. [31], 12 wild tomatoes and four close related outgroup species were located in 11 of them. Some of the 16 species of the genus *Solanum* showed specific patterns in their distribution within the identified climate types (Figure 1).

**Figure 2:**
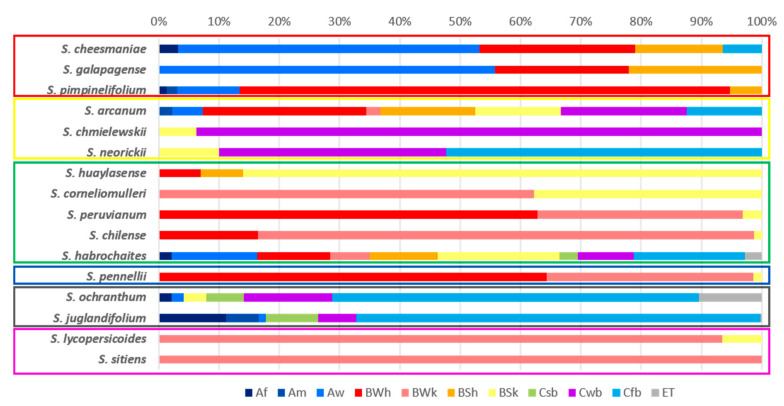
Percentage of climatic type by species according to Beck et al. [31] of 12 wild tomatoes (Sect. Lycopersicon) and 4 closely related species (Sect. Juglandifolia and Sect. Lycopersicoides). Climate classification: Af (tropical, rainforest), Am (tropical, monsoon), Aw (tropical, savannah), BWh (arid, desert, hot), BWk (arid, desert, cold), BSh (arid, steppe, hot), BSk (arid, steppe, cold), Csb (temperate, dry summer, warm summer), Cwb (temperate, dry winter, warm summer), Cfb (temperate, no dry season, warm summer), ET (polar, frost). Within each rectangle, species that belong to the phylogenetic groups proposed by Peralta et al. [1]: Red = Lycopersicon, yellow = Arcanum, green = Ericopersicon, blue = Neoricopersicon, gray = *S.* sect. *Juglandifolia*, pink = *S.* sect. *Lycopersicoides*.

The species that presented accessions in the greatest number of climates are *S. habrochaites*, *S. arcanum*, *S. ochranthum*, and *S. juglandifolum*. In contrast, the species with the highest environmental restrictions were *S. sitiens*, *S. lycopersicoides*, *S. corneliomulleri,* and *S. chmielewskii*. Regarding the diversity of climates, those with the greatest predominance among the species are BSk, BWk, and BWh, corresponding to climates of the cold steppe arid type, arid cold desert, and hot arid desert, respectively. Figure 2 shows the distribution and percentage of climates in each species. In this image, the climatic similarity between nearby taxonomic groups derived from phylogenetic data can be observed [1], identifying the same climate types in different proportions for species groups.

### 2.2. Ecological Descriptors

Ecological zones, as well as the distribution environments and altitudinal ranges reported in the literature for the 16 species of *Solanum* (Sect. Lycopersicon, Juglandifolia, and Lycopersicoides), are shown in Table 1. There were no specific reports of annual mean temperature, annual precipitation, mean diurnal range and annual evapotranspiration, or any other variable in the available literature. In some cases, some climatic parameters associated with the distribution zones are mentioned in general [1,22,23].

The ecological descriptors derived from the geographic location of the accessions and the EIS through the use of GIS tools are shown in Table 2. The variables considered were chosen due to their influence on the establishment of the species: altitude, annual mean temperature, mean diurnal range, annual precipitation, and annual evapotranspiration.

Both the ecological descriptors and the values reported by Peralta et al. [1] and Grandillo et al. [22] show very similar altitudinal ranges. Considering the median altitude values (Table 2) *S. cheesmaniae, S. galapagense,* and *S. pimpinelifollium* are the species with the lowest altitudinal range (45–93 m above sea level), and *S. lycopersicoides*, *S. sitiens*, and *S. ochranthum* are the species with the highest altitude range (2740–2928 m above sea level).

The annual mean temperature is a useful variable in the establishment and development of species. It influences, together with other biophysical variables such as relative humidity and precipitation, growth, and productivity of the species.

*S. cheesmaniae*, *S. galapagense*, and *S. pimpinelifollium* are distributed in areas where the median annual mean temperature is above 20 °C, while *S. lycopersicoides* and *S. sitiens* are the species distributed in sites with the lowest annual mean temperature value. The amplitude of the temperature changes, valued by the mean diurnal range, places *S. peruvianum* as the species that are located in places with less thermal oscillation; in contrast, *S. chilense* is located in places with greater thermal oscillation.

*S. juglandifolium* and *S. ochrantum* require the highest water requirements (annual precipitation and evapotranspiration); in contrast, *S. lycopersicoides* and *S. sitiens* are species characteristics of more arid and drier sites.

### 2.3. Statistical Analysis

**Figure 3:**
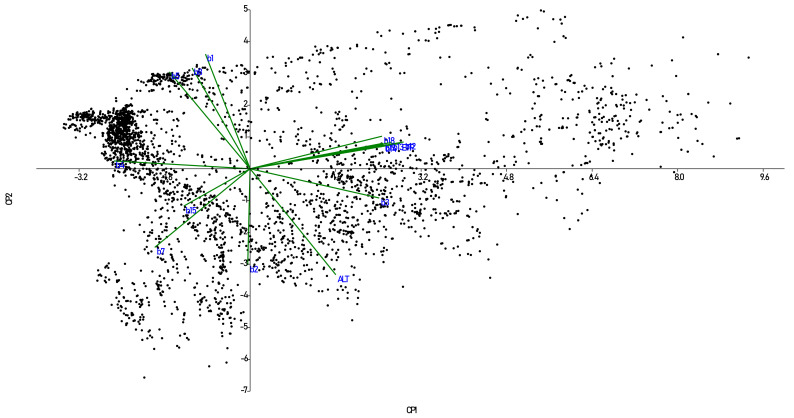
Biplot based on 4228 accessions of wild tomato and related species and 10 climatic variables. BIO13: precipitation of wettest month, BIO12: annual precipitation, ETPA: annual evapotranspiration, BIO1: annual mean temperature, BIO2: mean diurnal range, ALT: altitude, BIO8: mean temperature of wettest quarter, BIO5: maximum temperature of the warmest month, BIO15: coefficient of variation of seasonal precipitation, and BIO3: isothermality. PC1 and PC2 explained 47.8 and 27.2% of the total variation, respectively.

Linear correlation analysis detected multicollinearity between variables BIO6, BIO9-BIO11, BIO16, and BIO17, discarding them (10 variables) from subsequent statistical analyzes. The association patterns between variables were identified using a PCA. Thus, three principal components (PC1, PC2, and PC3) explained 86.2% of the variation, with an individual contribution of 47.8, 27.2, and 11.2%, respectively. Figure 3 shows the biplot of PC1 and PC2 (explaining 75% of the variation), showing the dispersion of the accessions and the contribution of the variables used. PC1 grouped variables related to water and humidity requirements: precipitation of wettest month (BIO13), annual precipitation (BIO12), and annual evapotranspiration (ETPA). PC2 was associated with annual mean temperature (BIO1), mean diurnal range (BIO2), altitude (ALT), mean temperature of the wettest quarter (BIO8), and maximum temperature of the warmest month (BIO5). Finally, PC3 was integrated by the coefficient of variation of seasonal precipitation (BIO15) and isothermality (BIO3).

**Figure 4:**
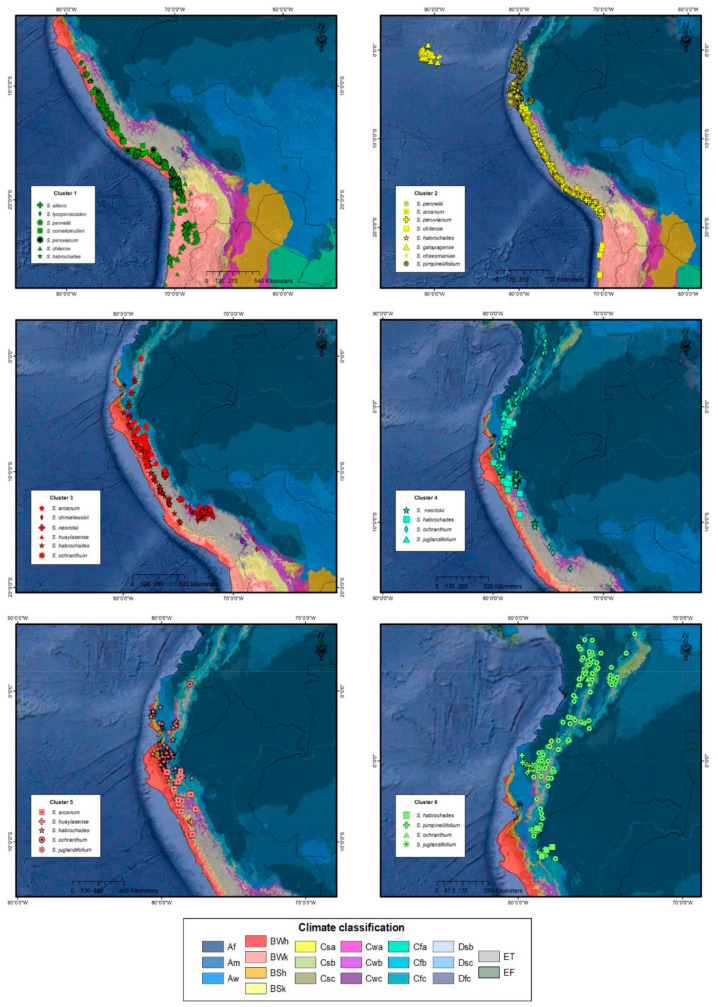
Clusters formed and their distribution of 12 wild tomatoes and 4 related species of *S. lycopersicum* based on climatic variables using Gower’s distances and Ward’s grouping method.

The CA was carried out in order to identify patterns of similarity between accession distribution areas. This analysis included the median values of informative variables previously selected for 67 combinations identified, resulting from the interaction of species by climate type, using the distances of Gower and Ward’s grouping method. According to the statistical indicators pseudo-F and pseudo t^2^, the number of statistically significant groups was six. In order to corroborate the belonging observations to each identified group, a discriminant analysis was carried out, where the test of restitution of linear discriminant function was applied, which did not indicate changes in the groups generated by the CA, confirming that the classification is reliable. The geographical distribution of the accessions that belong to each group is shown in Figure 4. Table 3 shows the medians and coefficients of variation of each of the groups identified in the CA. The significant variables of the PC were used to describe the groups generated in the CA. From these results, it is possible to identify ecological patterns among the groups formed; for example, the accessions of cluster 1 are those that are found at the highest altitude, and with the lowest annual mean temperature, the species that form cluster 4 and 6 are those with the highest annual precipitation and evapotranspiration, and species of group 1 are located in sites with less availability of humidity. Regarding Kruskal–Wallis non-parametric test of variables for obtained clusters, in all cases, the results were statistically significant (*p* ≤ 0.001). Table 4 shows the median values for each cluster, and the corresponding results of rank means comparison of informative variables of 3 PCs.

## 3. Discussion

Characterization of genetic resources through environmental information of accession sites, also called ecogeographic description, allows the typification of adaptive ranges and the most relevant environmental factors that determine species adaptation [24]. On the other hand, using GIS techniques, georeferencing species sites allows the analysis of geographical distances and distribution patterns of germplasm collection sites. With this approach, it is possible to determine environmental conditions in which the wild species and local varieties of crops have acquired their adaptive ranges [25]. This ecogeographic characterization complements the phenotypic and genetic information, useful for the characterization of the germplasm.

The distribution of the 67 combinations of species with climates in the clusters and the phylogenetic group to which they belong can be observed in Table 3. The Lycopersicon group, corresponding to the species of the Galapagos Islands and some continental areas, is located in clusters 2 and 6. *S. pennelli* (Neolicopersicon group) is located in clusters 1 and 2. The species of the Arcanum group and *S.* sect. *Juglandifolia* are located in four of the six proposed clusters. *S. huaylasense*, *S. corneliomulleri*, *S. peruvianum*, *S. chilense,* and *S. habrochaites*, species of the Ericopersicon group are distributed in all the clusters formed. On the other hand, *S.* sect. *Lycopersicoides* is only present in cluster 1.

Some species are distributed in a more restricted area (*S. lycopersicoides*, *S. sitiens*), and others are more widely located, a condition attributed to their wide distribution. It is worth mentioning that each species has a specific geographic distribution, with overlapping regions between various species, reflecting their ecological adaptation patterns and habitat preferences [22] (Figure 2).

Regarding the climatic diversity of the species of the genus *Solanum*, there were no concrete data in the literature for all the species considered within the tomato group. Thus, the diversity of climates, ecological descriptors, and abundance patterns described by this research for each species constitutes new and valuable information, with potential use for the identification of germplasm tolerant to specific adverse biotic and abiotic factors, among other purposes (Table 2, Table 3 and Table 4, Figure 2 and Figure 3). Wild tomato species are frequently found in isolated valleys with adaptations to particular types of climate, with possible tolerance or resistance to adverse conditions. Probably the Andean geography, the ecological diversity of habitats and climates together contributed to the diversity of wild species [32,33]. In general, it is mentioned that the wild tomato species are distributed in Ecuador, the Galapagos Islands, Peru, and the north of Chile and Colombia, in various ecosystems from sea level to approximately 3300 m above sea level [33,34,35]. It is important to highlight that a predictive classification of tomatoes and closely related groups were considered as a framework for the ecogeographic characterization and the actual taxonomic knowledge to select reliable species accessions from different sources of the database. This selection process is fundamental to generate a trustworthy species database for further analysis. There are often mistakes and inconsistencies due to incorrect taxonomic identification of accessions or wrong information of collection sites.

Few studies have been carried out with an ecogeographic or climatic approach, highlighting the publications of Peralta et al. [1], Nakazato et al. [22], Grandillo et al. [23], and Pease et al. [7]. These authors identified the ecological distribution environments and altitudinal ranges of adaptation of the tomato species. Both the ecological descriptors and the patterns of climatic diversity were generated from more current and more representative sources of information due to the diversity of variables of the EIS used and a large number of accessions from different sources of information. It should be noted that, although the results in altitude and ecological zones of distribution are very similar, the ecological descriptors provide information for the 16 species with greater amplitude and precision (Table 2 and Table 3).

With the information generated, it is also possible to begin to identify those species found in critical environments that can be potentially used as a source of germplasm for genetic breeding programs for resistance to drought [11], extreme temperatures [12], resistance to pests and diseases [13,14], to mention some examples.

There are specific studies of some species to which a certain adaptation or tolerance characteristic has been attributed due to their distribution; For example, *S. pennellii* is considered a species with extreme tolerance to drought attributed to strict control of transpiration, increased efficient use of water, and tolerance to soil salinity [36,37]; *S. sitiens* is considered the species that inhabits the most arid places [38] with the ability to tolerate high levels of salinity [22], *S. habrochaites* is known to have good growth at low temperatures [39,40,41], and *S. lycopersicoides* has resistance to drought and has a preference for colder sites [42,43]. These statements coincide with the values obtained in the ecological descriptors; for example, *S. sitiens* and *S. lycopersicoides* are located as the species from sites with the lowest availability of humidity.

In the present study, the use of multivariate analysis allowed to satisfactorily identify the climatic variables with the greatest association with the distribution of the ecogeographic diversity of the species. The present results, through the PCA, indicated associations among variables of altitude, humidity, and temperature, explaining in good proportion the variability of the data. Such behavior in the results satisfactorily summarizes the importance of the variables in the distribution of the *Solanum* species evaluated.

The characterization of the species generated from the CA could be satisfactorily validated by means of a discriminating analysis. In addition, some of the species that form the groups agree with the analysis of morphological and genetic characters, so it is possible to assume that there are relationships between these characters and the climatic characteristics generated, coinciding with the results of previous research [1].

An example of the validation of groups mentioned above is shown between *S. sitiens* and *S. lycopersicoides* considered as a group of related species or sister taxa by a cladistic study carried out by Peralta and Spooner [3] with morphological data and other similar investigations [1,44,45,46]. *S. neorickii* and *S. chmielewskii* are considered sister species [1], according to studies based on ITS sequences [47], analysis of phenotypic data and microsatellite markers [48], and cladistic studies with morphological data [49].

Conesa et al. [50] performed a climatic classification of 14 of the wild and related species to *S. lycopersicum* based on the mean value of annual precipitation and temperature and the De Martonne index. These authors proposed the formation of three groups: species from humid regions (*S. ochranthum*, *S. neorickii*, *S. chmielewskii*, *S. juglandifollium* and *S. lycopersicum*), species from semi-arid sites (*S. arcanum*, *S. habrochaites*, *S. pimpinelifollium*, *S. galapagense,* and *S. chesmaniae*) and species from arid regions (*S. sitiens*, *S. chilense*, *S. lycopersicoides*, *S. penneellii*, and *S. peruvianum*). This classification agrees with the results obtained from the mean annual precipitation reported in the ecological descriptors (Table 2).

The present study, in addition to identifying valuable ecogeographic information not previously reported in the literature, constitutes a precedent for investigating from the use of tools developed by GIS, collections and/or valuable distribution areas as a source of germplasm for the development of varieties tolerant and resistant to specific biotic and abiotic factors through genetic improvement.

In addition, this information can be used for the formation of germplasm conservation strategies, identification of material in danger of extinction due to climate change, and germplasm collection routes for the formation of core collections. Likewise, when addressing the classification of the ecogeographic conditions achieved, they could be associated with the presence of adverse factors, both biotic and abiotic, to define areas with the probable presence of genes for resistance to such factors. Finally, it is important to mention that it is necessary to identify the actual and future ecological niches of the studied species, in that sense, these results constitute the first step on the ecogeography of the wild tomato species, being necessary to identify the ecological niches and the impact of climate change on their distribution and ecological patterns.

## 4. Materials and Methods

### 4.1. Database

Passport data of georeferenced accessions of 12 species of wild tomatoes (*S.* sect. *Lycopersicon*) and 4 species of phylogenetically related groups (*S.* sect. *Juglandifolia* and *S.* sect. *Lycopersicoides*) were used. A database was built with information from scientific reports and articles [36,51,52,53,54,55] and national (World Biodiversity Information Network) [56] and international plant inventories (Tomato Genetics Resource Center, Global Biodiversity Information Facility, Solanaceae source) [21,57,58].

It was possible to collect the coordinates of 11,707 accessions, which were reviewed to rule out atypical data, eliminating repeated records, with coordinates of little geographic precision (less than 3 decimal places) and accessions outside the study area according to altitude reported and respecting the previously distributed areas described by Peralta et al. [1] and Grandillo et al. [23] (Table 1). All these strategies were applied to avoid considering accessions that correspond to introductions outside the natural areas of distribution. Finally, a frequency analysis was applied, eliminating those accessions associated with climatic types with less than 3 accessions. From this, 4228 accessions of 12 wild tomatoes and 4 closely related species distributed in Latin America were selected (Figure 1).

### 4.2. Environmental Information

The EIS was built with 21 variables included annual evapotranspiration (EVAPO, mm) and site altitude (ALT, m), as well as the bioclimatic variables from WorldClim version 2.1 (1970–2000) with spatial resolution of ~1 km^2^ [59]: annual mean temperature (BIO1, °C), mean diurnal range (BIO2, °C), isothermality (BIO3), seasonal temperature (BIO4), maximum temperature of warmest month (BIO5, °C ), minimum temperature of coldest month (BIO6, °C), temperature annual range (BIO7, °C), mean temperature of wettest quarter (BIO8, °C), mean temperature of driest quarter (BIO9, °C ), mean temperature of warmest quarter (BIO10, °C), mean temperature of coldest quarter (BIO11, °C), annual precipitation (BIO12, mm), precipitation of wettest month (BIO13, mm), precipitation of driest month (BIO14, mm), precipitation seasonality (BIO15), precipitation of wettest quarter (BIO16, mm), precipitation of driest quarter (BIO17, mm), precipitation of warmest quarter (BIO18, mm) and precipitation of coldest quarter (BIO19, mm).

Annual evapotranspiration was calculated from monthly values in raster format with a spatial resolution of 30 arcs second (~1 km^2^) [60]. Finally, the altitude of the collection site of each accession was determined from an elevation model in raster format, also with spatial resolution ~1 km^2^ [61].

Climatic types of the accession sites were defined from the world climatic classification with the Köppen–Geiger system [62] with a spatial resolution of ~1 km^2^ proposed by Beck et al. [31]: Af, Am, Aw, BWh, BWk, BSh, BSk, Csa, Csb, Csc, Cwa, Cwb, Cwc, Dsa, Dsb, Dsc, Dsd, Dwa, Dwb, Dwc, Dwd, Dfa, Dfb, Dfc, Dfd, ET, and EF.

### 4.3. Climatic Diversity and Ecological Descriptors

Climatic diversity patterns were identified with vectors of the geographical location of each accession. With these vectors and the “Extraction” module of the ArcGis software “Spatial Analyst Tools”, the value of each pixel of the corresponding climatic classification was considered, then all the information was integrated into a worksheet (Microsoft Excel) to identify all types and frequencies of climates for each species.

Ecological descriptors were determined with the methodology proposed by Ruiz-Corral et al. [11], using geographic location vectors of all accessions and the EIS; with this, climatic ranges of adaptation were identified. These values were obtained with the ArcGis “Spatial Analyst Tools”. Information was concentrated in a worksheet where extreme (minimum and maximum) and median and coefficient of variation of each variable for each species were subsequently determined [10,27].

### 4.4. Statistical Analysis

Linear correlations between pairs were obtained to identify multicollinearity between variables. In those variables with an absolute coefficient greater than 0.95, one of the corresponding pairs was chosen. With the chosen variables, a PCA was carried out to identify the most important variables in the description of the variation between accessions. In order to identify the similarity between the species from the present climatic diversity, a grouping analysis (CA) was carried out with the Gower distances and Ward’s method of minimum variance. In order to carry out this analysis, the possible combinations between species (16) and climatic type (11) were identified, with which 67 combinations were obtained. To corroborate the belonging observations to each identified group, a discriminant analysis was carried out. Finally, the non-parametric test Kruskal–Wallis and range comparison test [63] were performed for clusters generated. To describe the groups, the variables identified as significant in the PCA were used. Statistical analyses were carried out using the Statistical Analysis System software version 9.4 [64].

## Figures and Tables

**Table 1:** Distribution and altitude (m above sea level) of 16 species of *Solanum* reported in two publications: Peralta et al. [1] and Grandillo et al. [23]. Species described according to taxonomic sections and groups proposed by Peralta et al. [1].

Section/Group	*Solanum* Species	Ecological Distribution	Altitude [1]	Altitude [23]
Section Lycopersicon Lycopersicon	*S. cheesmaniae*	Endemic to the Galapagos Islands. It inhabits dry, open, and rocky slopes, cold places.	0–1300	0–1500
	*S. galapagense*	Endemic to the Galapagos Islands, on dry, open, and rocky slopes.	0–600 (1500)	0–650
	*S. pimpinellifolium*	Southern region of Ecuador to the northern region of Chile. Dry slopes, plains, and associated with crop areas.	0–500	0–500
Arcanum	*S. arcanum*	Northern Peru in inter-Andean dry valleys and coastal ecosystems with seasonal fog. Generally dry sites, rocky slopes.	100–2500	500–3000
	*S. chmielewskii*	Southern zone of Peru and the northern zone of Bolivia. Wet and well-drained rocky slopes.	2300–3000	1600–3100
	*S. neorickii*	Southern Ecuador to southern Peru, in inter-Andean dry valleys.	1950–3000	1500–2500
Ericopersicon	*S. huaylasense*	Northern and central Peru, on dry, open, and rocky slopes.	1700–000	1000–900
	*S. corneliomulleri*	Southern Peru in regions with dry and rocky slopes.	1000–3000	1000–3000
	*S. peruvianum*	Central region of Peru to northern Chile in dry coastal deserts and seasonal mist ecosystems.	0–600	0–2500
	*S. chilense*	Coastal zone of Chile and northern Peru, on dry rocky slopes, and occasionally saline.	0–3000	50–3500
	*S. habrochaites*	Andean region of Ecuador and Peru in montane forests and dry slopes, occasionally found in seasonal fog ecosystems.	400–3600	40–3300
Neolicopersicon	*S. pennellii*	North of Peru to the north of Chile, in areas of dry slopes, generally in flat areas.	0–3000	0–1920
Section Juglandifolia	*S. ochranthum*	Andean region of Colombia, Ecuador, and Peru, areas of mountain mesophilic forest.	1900–4100	1200–3200
	*S. juglandifollium*	Andean region of Colombia, Ecuador, and Peru in areas of mountain mesophilic forest.	1200–3100	1200–3100
Section Lycopersicoides	*S. lycopersicoides*	Southern area of Peru and northern Chile. In ravines and rocky slopes.	1500–3700	1200–3700
	*S. sitiens*	Hyper-arid areas, northern region of Chile.	2350–3500	2500–3500

**Table 2:** Ecological descriptors of the wild and related species to *S. lycopersicum*. ALT = altitude, TEMP = annual mean temperature, DRAN = mean diurnal range, RAIN = annual precipitation, EVAPO = annual evapotranspiration. Max = maximum, Min = minimum, Med = median, and CV = coefficient of variation. Species divided according to sections and groups proposed by Peralta et al. [1].

Sections/Group	Species	ALT				TEMP				DRAN				RAIN				EVAPO			
		(m)				(°C)				(°C)				(mm)				(mm)			
		Max	Min	Med	CV	Max	Min	Med	CV	Max	Min	Med	CV	Max	Min	Med	CV	Max	Min	Med	CV
Section Lycopersicon Lycopersicon	*S. cheesmaniae*	1478	5	87	155.20	25	17.1	23.6	3.27	10.3	7.6	8.5	4.57	562	107	277	21.70	1125	187	454	42.24
	*S. galapagense*	868	4	45	240.00	25	19.8	23.9	2.87	10.1	7.6	8.7	4.91	546	135	274	16.06	930	262	531	30.60
	*S. pimpinelifollium*	633	1	93	100.00	26.8	17.9	22.7	8.54	13.3	6.3	9.8	8.37	2989	1	70	157.86	1710	1	45	202.22
Arcanum	*S. arcanum*	3292	132	1681	36.76	24.1	11.4	18.4	9.19	14.4	10.3	12.4	3.42	1193	22	527	43.45	1094	11	395	43.29
	*S. chmielewskii*	3195	1953	2583	9.52	19.9	13	16.8	9.88	15.6	11.7	14.9	3.21	1318	504	944	18.51	874	429	647	16.74
	*S. neorickii*	3262	1705	2317	11.57	20.3	11.7	16.9	7.88	15.5	9.9	13.1	2.19	1366	426	816	23.53	1031	326	672	14.29
Ericopersicon	*S. huaylasense*	3124	1141	2291	16.06	20.3	11.2	16.8	8.38	13.9	10.8	13.4	1.27	500	128	346	23.99	416	73	278	30.76
	*S. corneliomulleri*	3097	1018	2344	18.75	18.3	9.6	14.1	12.95	16.6	9.4	12.3	4.07	434	19	201	60.70	354	12	141	48.58
	*S. peruvianum*	2617	2	528	124.03	20.9	11.7	18.6	6.98	15.5	4.7	9.4	15.61	434	0	25	92.00	324	0	13	130.77
	*S. chilense*	3995	0	1904	59.53	20.4	5.4	15.2	19.29	18.6	4.9	12.6	10.82	355	0	28	69.64	275	3	20	72.50
	*S. habrochaites*	3692	40	2033	33.78	25.8	7	16.6	17.30	14.5	6.5	11.8	5.26	2358	11	622	43.25	1682	8	555	43.69
Neolicopersicon	*S. pennellii*	2921	5	822	53.16	25.1	10.5	18.4	7.38	13.7	6.2	10.2	8.35	404	1	49	95.92	289	0	33	96.97
Section Juglandifolia	*S. juglandifolium*	3353	1005	2195	14.49	22.9	8.9	15.8	10.68	12.5	7.2	9.1	1.94	3214	550	1895	28.79	1648	413	1177	10.24
	*S. ochranthum*	4008	1195	2750	10.27	21.9	6.7	13.9	11.65	15.6	7.2	11.4	3.42	2358	507	1010	11.44	1474	286	818	13.57
Section Lycopersicoides	*S. lycopersicoides*	3775	1290	2928	13.54	17.3	7.6	11.2	15.28	15.1	10.4	14.1	4.55	215	13	104	53.14	182	9	80	60.94
	*S. sitiens*	3330	2276	2740	5.90	13.2	8.4	11.4	7.94	17.6	15.6	16.8	1.26	31	8	17	25.00	26	9	21	16.67

**Table 3:** Median (Med) and coefficient of variation (CV) of the ecological descriptors of the combinations of 16 wild and related tomato species by the 11 climatic types identified according to the formation of CA groups. Group = A (Arcanum), E (Ericopersicon), N (Neolicopersicon), Y (Section Lycopersicoides), L (Lycopersicon), and J (Section Juglandifolia). Spe = species, Clim = climate type [31], ALT = altitude, TEMP = annual mean temperature, DRAN = mean diurnal range, RAIN = annual precipitation, EVAPO = annual evapotranspiration. JUG = *S. juglandifolium*, OCH = *S. ochranthum*, CHI = *S. chilense*, COR = *S. corneliomulleri*, HAB = *S. habrochaites*, HUA = *S. huaylasense*, LYC = *S. lycopersicoides*, PEN = *S. pennelli*, ARC = *S. arcanum*, CHM = *S. chmielewskii*, NEO = *S. neorickii*, CHE = *S. cheesmaniae*, GAL = *S. galapagense*, PIM = *S. pimpinelifollium*, PER = *S. peruvianum*, SIT = *S. sitiens*. Climate types: 1 = Af (tropical, rainforest), 2 = Am (tropical, monsoon), 3 = Aw (tropical, savannah), 4 = BWh (arid, desert, hot), 5 = BWk (arid, desert, cold), 6 = BSh (arid, steppe, hot), 7 = BSk (arid, steppe, cold), 9 = Csb (temperate, dry summer, warm summer), 12 = Cwb (temperate, dry winter, warm summer), 15 = Cfb (temperate, no dry season, warm summer), 29 = ET (polar, frost).

Group	CLUTER	Spe-Clim	ALT (msnm)		TEMP (°C)		DRAN (°C)		RAINF (mm)		EVAPO (mm)	
			Med	CV	Med	CV	Med	CV	Med	CV	Med	CV
E	1	CHI-5	2280	44.52	14.1	20.36	13.1	16.98	36	54.17	26	59.62
E	1	CHI-7	3662	12.56	8.1	26.39	14.8	8.26	238	16.81	208	13.73
E	1	COR-5	1989	22.08	15.6	6.65	12	13.81	130	46.15	94	36.9
E	1	COR-7	2637	7.47	11.7	8.4	12.3	2.2	360	9.17	239	15.9
E	1	HAB-29	3558	3.11	9.2	3.38	13.4	4.23	562	1.6	480	18.13
E	1	HAB-5	1805	9.17	15.8	7.66	11.6	2.48	194	16.49	137	28.1
Y	1	LYC-5	2881	13	11.6	13.83	14	4.78	93	59.68	76	61.18
Y	1	LYC-7	3654	0	8.1	0	14.9	0	213	0	182	0.27
N	1	PEN-5	1531	16.98	16.7	4.53	11	6.64	90	56.11	75	46.67
N	1	PEN-7	2526	8.22	12.8	15.18	12.6	1.73	335	11.79	250	14.83
E	1	PER-5	1713	29.19	16	7.72	11.6	12.98	55	104.55	51	77.45
E	1	PER-7	2472	4.69	12.9	3.03	12.3	3.73	339	12.24	213	17.84
Y	1	SIT-5	2740	5.9	11.4	7.94	16.8	0.95	17	25	21	16.67
A	2	ARC-4	679	38.25	20	2.3	11.5	5.29	91	101.1	84	108.33
L	2	CHE-1	620	13.43	21.3	3.6	8.7	8.23	305	7.46	988	6.4
L	2	CHE-15	1013	10.27	19.7	2.61	10	2.72	328	4.81	992	4.39
L	2	CHE-3	144	79.44	23.4	2.12	8.2	4.8	275	16	457	38.29
L	2	CHE-4	47	63.98	24.3	0.69	8.9	1.66	202	22.4	290	7.67
L	2	CHE-6	39	108.97	24.1	1.24	8.4	2.54	359	39.28	504	12.81
E	2	CHI-4	385	90.78	18.7	2.31	9	20.18	7	71.43	7	21.43
L	2	GAL-3	167	81.14	23.4	2.71	8.6	4.7	276	17.93	562	29.36
L	2	GAL-4	20	77.5	24.4	1.27	9.1	4.32	249	18.07	322	39.91
L	2	GAL-6	18	83.33	24.4	0.52	9.3	5.1	275	11.27	531	19.68
E	2	HAB-4	326	110.43	20.6	9.33	11.3	6.28	114	57.46	77	92.86
N	2	PEN-4	622	43.09	19.6	4.5	10	8.15	34	88.24	19	73.68
L	2	PIM-3	27	414.81	25.2	1.42	9.1	5.01	1190	35.88	861	12.31
L	2	PIM-4	95	87.37	22	8.03	10.1	12.63	50	119	25	224
L	2	PIM-6	64	276.38	24.7	2.58	9.8	4.61	602	14.29	485	15.77
A	3	ARC-12	2373	9.59	16.3	6.04	13.6	2.39	716	8.1	594	9.6
A	3	ARC-15	2115	13.36	16.8	6.28	12.3	2.43	863	6.55	745	7.99
A	3	ARC-7	2537	17.03	15.1	11.98	13.4	2.46	352	34.23	370	13.24
A	3	CHM-12	2601	9.88	16.7	10.31	15	1.92	948	14.45	663	14.25
A	3	CHM-7	2560	4.02	17.1	4.81	14.3	1.66	534	17.88	449	24.28
E	3	HAB-12	2692	9.68	14.8	9.02	13.7	8.13	761	8.54	645	7.67
E	3	HAB-7	2648	10.84	13.4	12.73	12.9	4.96	393	21.12	296	32.6
E	3	HUA-7	2337	9.99	16.6	5.51	13.6	2.67	354	20.06	282	28.19
A	3	NEO-12	2564	10.26	16.8	9.75	14.6	5.17	999	27.18	626	16.37
A	3	NEO-7	2096	11.09	18.7	8.45	13.8	2.15	541	7.95	419	24.94
J	3	OCH-12	3010	11.05	13.8	10.51	14.4	4.62	967	17.12	664	14.31
J	3	OCH-7	2865	10.12	15	4.04	15.2	10.95	508	25	442	3.73
E	4	HAB-15	2270	8.37	16.1	5.65	11.6	7.69	921	13.74	799	12.52
E	4	HAB-9	2733	12.22	13.9	8.39	11	1.02	595	2.69	582	10.82
J	4	JUG-12	2428	7.91	13.7	4.34	10	6.12	760	18.42	778	8.74
J	4	JUG-29	3144	0	10.9	0	10.7	0	1222	0	1,015	0
A	4	NEO-15	2153	11.18	16.9	6.68	11.8	4.17	817	15.09	719	7.82
J	4	OCH-15	2626	9.12	14	9.08	11.2	8	1041	10.25	825	13.58
J	4	OCH-29	3372	4.09	9.1	6.24	9.9	7.99	1010	6.76	812	11.02
J	4	OCH-9	2725	5.42	13.8	5.12	10.9	6.91	1004	10.48	932	9.87
A	5	ARC-2	1216	0	21.4	0	11.1	0	1193	0	1094	0
A	5	ARC-3	1448	6.01	19.9	2.25	12.2	0.31	694	5.69	537	4.75
A	5	ARC-5	1828	6.44	17.3	2.77	12.2	4.99	232	27.05	179	42.58
A	5	ARC-6	1259	13.15	19.4	5.59	12.5	3.21	453	22.02	358	24.23
E	5	HAB-3	1415	20.61	20.7	7.82	11.7	5.38	918	13.73	840	12.56
E	5	HAB-6	1077	32.4	21.3	7.65	11.7	3.77	566	18.73	479	18.37
E	5	HUA-4	1234	15.36	19.3	2.65	12.4	7.72	247	32.79	194	34.54
E	5	HUA-6	1477	19.77	19.3	3.66	12.6	3.35	304	8.22	215	7.44
J	5	JUG-3	1510	9.3	18.4	3.41	10.2	3.23	1,099	18.15	737	23.0
J	5	OCH-3	1734	6.31	19.2	3.69	11.7	4.15	860	6.89	867	14.95
E	6	HAB-1	629	51.55	24.2	3.79	10.9	2.06	1708	9.69	1549	8.78
J	6	JUG-1	1515	10.73	19.7	5.06	9.6	6.74	2274	9.8	1363	6.64
J	6	JUG-15	2275	9.78	15.4	8.23	9.1	7.35	1941	27.79	1183	8.54
J	6	JUG-2	1325	8.42	19.6	3.03	8.6	2.76	2087	8.53	1189	3.36
J	6	JUG-9	1933	12.78	16.6	6.31	9.0	5.15	1456	18.37	1148	3.53
J	6	OCH-1	1472	10.09	19.9	4.57	9.9	5.47	2009	10.63	1333	12.87
L	6	PIM-1	379	41.56	25.5	3.5	10.4	6.78	2080	20.82	1655	15.38
L	6	PIM-2	289	60.14	24.3	3.52	8.2	1.62	2494	17.3	1221	5.2

**Table 4:** Medians comparisons of the informative variables that integrate the first three principal components for the 6 clusters formed in the CA of the wild tomato species in Latin America. EVAPO = Annual evapotranspiration, BIO12 = annual precipitation, BIO13 = precipitation of wettest month, ALT = altitude, BIO8 = mean temperature of wettest quarter, BIO5 = maximum temperature of warmest month, BIO2 = mean diurnal range, BIO1 = annual mean temperature, BIO15 = precipitation seasonality, and BIO3 = isothermality.

	CP1			CP2					CP3	
	EVAPO	BIO12	BIO13	ALT	BIO8	BIO5	BIO2	BIO1	BIO15	BIO3
1	137 d	194 d	69 c	2526 b	14.0 cd	20.5 d	12.6 b	12.8 d	127 a	74.6 c
2	389 c	262 d	54 c	155 d	25.1 a	30.0 a	9.0 e	22.7 a	89 b	67.7 d
3	521 c	628 c	117 b	2562 ab	16.5 c	24.0 c	13.7 a	16.4 c	80 c	84.1 b
4	805 b	962 b	141 b	2675 a	13.9 d	20.7 d	10.9 d	13.8 cd	43 d	85.0 ab
5	508 c	630 c	129 b	1431 c	20.3 b	26.0 b	11.9 c	19.3 b	92 bc	86.6 a
6	1277 a	2044 a	253 a	1398 c	19.8 b	25.4 b	9.3 e	19.7 ab	35 d	88.9 a

Medians with the same letter within each column are not statistically different (*p* ≤ 0.05) according to the multiple comparisons and Kruskal–Wallis test.

## Data Availability

All data sources supporting accessions and climatic information used are mentioned at Methodology section: Solanaceae Source (http://solanaceaesource.org/, accessed on 15 December 2020), GBIF (https://www.gbif.org/, accessed on 19 December 2020), TGRC (https://tgrc.ucdavis.edu/, accessed on 19 December 2020) WorldClim (https://www.worldclim.org/, accessed on 10 December 2020).
